# Fast prediction of personalized abdominal organ doses from CT examinations by radiomics feature-based machine learning models

**DOI:** 10.1038/s41598-024-70316-7

**Published:** 2024-08-20

**Authors:** Wencheng Shao, Xin Lin, Wentao Zhao, Ying Huang, Liangyong Qu, Weihai Zhuo, Haikuan Liu

**Affiliations:** 1 Institute of Radiation Medicine, Fudan University, Shanghai, China; 2 Department of Nuclear Science and Technology, Institute of Modern Physics, Fudan University, Shanghai, China; 3 Department of Radiology, Shanghai Zhongye Hospital, Shanghai, China

**Keywords:** Abdominal CT scanning, Patient-specific modeling, Radiation dosage, Radiomics, Support vector regression, Information theory and computation, Risk factors, Computational science, Scientific data

## Abstract

The X-rays emitted during CT scans can increase solid cancer risks by damaging DNA, with the risk tied to patient-specific organ doses. This study aims to establish a new method to predict patient specific abdominal organ doses from CT examinations using minimized computational resources at a fast speed. The CT data of 247 abdominal patients were selected and exported to the auto-segmentation software named DeepViewer to generate abdominal regions of interest (ROIs). Radiomics feature were extracted based on the selected CT data and ROIs. Reference organ doses were obtained by GPU-based Monte Carlo simulations. The support vector regression (SVR) model was trained based on the radiomics features and reference organ doses to predict abdominal organ doses from CT examinations. The prediction performance of the SVR model was tested and verified by changing the abdominal patients of the train and test sets randomly. For the abdominal organs, the maximal difference between the reference and the predicted dose was less than 1 mGy. For the body and bowel, the organ doses were predicted with a percentage error of less than 5.2%, and the coefficient of determination (R^2^) reached up to 0.9. For the left kidney, right kidney, liver, and spinal cord, the mean absolute percentage error ranged from 5.1 to 8.9%, and the R^2^ values were more than 0.74. The SVR model could be trained to achieve accurate prediction of personalized abdominal organ doses in less than one second using a single CPU core.

## Introduction

Abdominal diseases are hazards to people worldwide because they cause symptoms and complications in abdominal organs and tissues. According to the WHO's Global Health Estimates liver diseases caused 1,312,480 (4.6%) of 28,444,814 deaths in the Asia-Pacific region^1^ Irritable bowel syndrome (IBS), gastroesophageal reflux disease (GERD), and functional dyspepsia account for 14% to 45% of the prevalence of functional gastrointestinal disorders, depending on diagnosis criteria or region^2,3^. About 20% of people suffer from infectious intestinal diseases in the United Kingdom each year, and serious complications (i.e., dehydration, malabsorption, sepsis, etc.) could also happen for those people^4^. Besides, stomach tumors have a relatively high incidence and may cause deadly complications or even deaths. In 2020, the estimated new stomach cancer cases and deaths respectively were 1.03 million and 782,685^5^. Thus, it is necessary to diagnose abdominal diseases as early as possible to make treatments more effective and enhance patients’ quality of life.

Computed tomography (CT) is widely applied in diagnosing abdominal diseases because of its ability to provide slice-wise anatomical images of abdominal organs and tissues. CT imaging could detect various abdominal diseases including infections, traumas, inflammations, hemorrhages, etc. For general surgery, CT imaging almost detects 95% of ordinary abdominal disease cases^6^. In the emergency department, 50% to 60% of patients with abdominal pain get examined by CT^7^. The radiation exposure from such a huge amount of abdominal CT imaging could be a risk factor that leads to secondary cancers in the long term. The data from the American Cancer Society (ACS) indicates a CT scan of the abdomen and pelvis makes a patient get as much as 10 mSv X-ray radiation^8^. This amount of radiation dose is almost equivalent to 3 years of natural background exposure. Australian retrospective research concluded that a 24% increase in overall cancer incidence was observed among those individuals who get exposed to CT radiation^9^. Thus, it is urgent to investigate the personalized organ dose from abdominal CT imaging so that the radiation-induced risk and potential complications can be accurately evaluated and controlled.

To date, size-specific dose estimate (SSDE) and Monte Carlo (MC) dose calculation, are the main methods for estimating personalized organ dose from CT diagnosis^10–19^. SSDE just considers size information but neglects the information regarding anatomy, tissue, and geometric shape, which may cause higher errors. MC simulation could calculate personalized organ dose directly from the patient’s CT and segmented regions of interest (ROIs) accurately, but it requires intensive computational resources of the central processing unit (CPU), graphics processing unit (GPU), or even in computing time depending on the MC software used. Until now, there were almost no previous studies that predicted personalized abdominal organ doses by training SVR models. Thus, it is necessary to explore new methods to accurately predict personalized abdominal organ doses from CT examinations within a short time using fewer computational resources.

In this study, the robust SVR model^20^ was trained based on the abdominal radiomics features and GPU MC-calculated organ doses to predict personalized abdominal organ dose within a short time, with relatively better robustness, using fewer abdominal patient cases and minimized computational resources. The abdominal radiomics features were extracted from auto-segmented organ contours to consider much more intensity, anatomical, and tissue properties beyond the water-equivalent diameter (D_w_) or patient size used by the SSDE prediction strategy. Abdominal organ contours were generated by auto-segmentation which was approved to be effective to accurately delineate internal organ contours^21^.

The SVR model trained based on radiomics features sufficiently reflected the correlation between complex abdominal patient features and organ doses, thus precisely predicting personalized abdominal organ doses. The performance of the organ dose estimation method was evaluated by calculating the relative root mean squared error (RRMSE), mean absolute percentage error (MAPE), and coefficient of determination (R^2^) on the test sets. The robustness of the SVR model was verified by randomly allocating patient samples to the train set and test set 20 times when keeping the proportion between the two sets as 0.8 versus 0.2.

### Ethics

The study was conducted following the Declaration of Helsinki. Informed consents was obtained from patients who underwent abdominal CT scans for diagnosing purpose. We used the existing CT data in our hospital to achieve retro research. The research was approved by the Ethics Committee of Shanghai Zhongye Hospital to access the CT data for research purposes. The study followed the ethical guidelines of the hospital. The CT data were anonymized, and no personal information was disclosed.

## Results

### Predicted organ doses

In this section, the mean values of reference and predicted abdominal organ doses and their corresponding standard deviation (SD) were computed on the test sets. As shown in Table 1, in terms of the mean organ doses on the test sets, the predicted organ doses fitted very well with the reference organ doses, the maximal difference between predicted and reference doses was smaller than 1 mGy. This indicated that the SVR model trained based on the abdominal radiomics features could achieve a relatively small deviation between the predicted and reference dose values.

**Table 1:** Mean reference and predicted abdominal organ doses on the test sets.

Organ	Mean reference dose (mGy)	SD of reference dose (mGy)	Mean predicted dose (mGy)	SD of predicted dose (mGy)
Body	31.06	10.01	30.83	9.45
Bowel	25.52	11.39	25.16	10.56
Kidney_L	26.73	9.73	26.27	8.62
Kidney_R	25.68	10.29	26.02	8.34
Liver	29.54	13.34	29.69	9.73
Spinal cord	16.84	7.78	17.06	5.56

In Table 1, the “KidneyL” and “KidneyR” respectively denote the left kidney and right kidney.

### Regression metrics for organ dose prediction

The RRMSE, MAPE, and R^2^ were calculated for the patient samples on the test sets. As exhibited in Table 2, the SVR prediction achieved the best performance as the RRMSE, MAPE, and R^2^ of 4.54%, 2.24%, and 0.96 for the body. For the abdominal organs except the body, the RRMSE values ranged from 7.34% to 12.78%, while the MAPE values were smaller than 8.89%. For all the investigated organs, the R^2^ values were more than 0.74 and reached up to the maximal value of 0.96 for the body.

**Table 2:** Calculated regression metrics on the test sets.

Organ	RRMSE (%)	MAPE (%)	R^2^
Body	4.54	2.24	0.96
Bowel	7.34	5.15	0.90
Kidney_L	10.12	5.94	0.79
Kidney_R	10.90	7.35	0.77
Liver	12.78	8.89	0.74
Spinal cord	9.17	8.63	0.75

In Table 2, the “KidneyL” and “KidneyR” respectively denote the left kidney and right kidney.

### Robustness of the SVR dose prediction model

**Figure 1:**
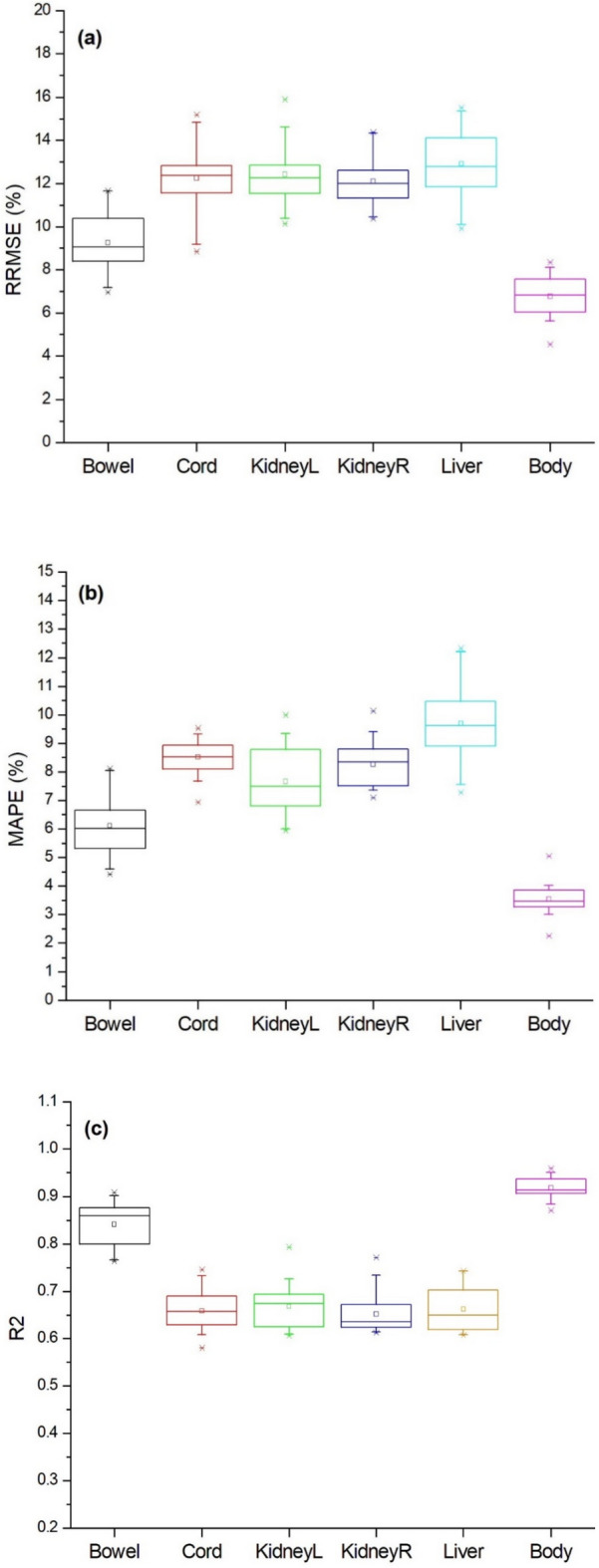
Box charts for RRMSE (**a**), MAPE (**b**), and R^2^ (**c**) for 20 random patient allocation strategies. In Fig. 2, the “KidneyL” and “KidneyR” respectively denote the left kidney and right kidney.

In this section, the patient samples on the training and test sets were randomly allocated 20 times keeping the proportion of the two sets as 0.8 versus 0.2. The regression metrics of the 20 patient allocation strategies were shown in Fig. 1. As seen in Fig. 1a, the interquartile range of RRMSE was from 1 to 2.5% for the abdominal organs, indicating that the performance of the SVR model remained stable when the patient samples were randomly rearranged. As illustrated in Fig. 1b, the interquartile range of MAPE was smaller than 2.2%. As shown in Fig. 1c, the interquartile range of R^2^ was less than 0.07%, which suggested that the performance of the SVR model remained stable against the variations in patient sample allocation strategies.

## Discussion

Abdominal CT imaging plays an essential role in detecting abdominal diseases, monitoring abdominal disease progression, and monitoring treatment outcomes. However, the ionization radiation from abdominal CT scans could cause radiation complications or even secondary cancers. This study aimed to explore a novel method to predict accurate and robust personalized abdominal organ doses when the resources for prediction were minimized, even minimized down to a single CPU core. Previous studies merely considered the patient size and some other simple features. Unlike existing studies, this study adopted the radiomics features to consider the characteristics of patient tissue, anatomy, and geometric shape, and train the SVR model for predicting personalized abdominal organ doses. The MAPE and R^2^ of the SVR regression model were about 0.08 and 0.7, respectively, which suggested that radiomics features could reflect the abdominal organ features (e.g. geometric shape, size, tissue, and anatomy) as much as possible.

There were already previous studies investigating personalized organ doses for different diagnosed sites (i.e. head, chest). However, studies using the SVR prediction model dedicated to abdominal CT examinations were very rare. For the previous studies that adopted SSDE, neural networks, convolutional networks, etc., the robustness of those models had not been validated by randomly assigning patient samples to the train and test sets. For the previous MC simulation studies, a massive amount of computing resources of multiple CPUs or GPUs were required, even professional computer science was one of the prerequisites to use MC simulation to calculate personalized organ doses. The trained SVR model just needs a simple Python script to predict organ doses in one second, with minimized computing resources, even with a single CPU. In practical abdominal CT examinations, the proposed method can be applied in two main ways. First, the method can be utilized immediately after CT reconstruction through three steps: importing the CT images into segmentation software (e.g., 3D Slicer) for abdominal organ delineation, extracting radiomics features quickly, and performing fast SVR-based prediction of abdominal organ doses for clinical organ dose evaluation. Second, the method can be applied to a large amount of retrospective abdominal CT data to enable fast batch prediction of abdominal doses for research purposes. The predictive performance in MAPE for previous studies with deep learning is from 2 to 10%, which is in the similar range with the performance of SVR-based abdominal organ dose prediction models. This indicates the proposed SVR-based models could achieve accurate and fast predict using much less computation resources compared with deep learning models. This valuable advancement could significantly reduce computational costs in terms of time, hardware, and electrical energy when predicting organ doses from ionizing radiation examinations on extremely large datasets for research purposes. We also compared the performance of SVR and fully connected neural networks (FCNN) in predicting chest organ doses from CT scans. For the right lung, left lung, overall lungs, esophagus, heart, and trachea, the trained SVR model achieved a MAPE between 0.10 and 0.18, with R-squared values ranging from 0.75 to 0.89 on the test sets. In comparison, the trained FCNN model had a MAPE ranging from 0.11 to 0.15 and R-squared values from 0.74 to 0.86 on both the training and test sets.

This study has several defects as well. First, we just trained the SVR prediction model from the abdominal radiomics features of the patients diagnosed in our institution. The accuracy and robustness of the model could vary among institutions. In the future, the collaboration of multiple institutions is still necessary to verify the cross-institution effectiveness of the proposed method. Second, the GPU-calculated reference organ doses were merely validated by CTDI_vol_ of the CT device in our institution. It is still necessary to train the SVR model for multiple CT device types. Although we investigated most of the abdominal organs, we did not include the stomach. The gastric contents varied severely among abdominal patients, causing PyRadiomics to be pretty hard to extract effective radiomic features. This variation hindered the ability to distinguish patient-specific characteristics between different patients. In the future, it is necessary to investigate the prediction model that has the potential to obtain more detailed image information inside the stomach. This study adopted resampling in Pyradiomics module to decrease the impact of voxel size discrepancy among different patients on standardized feature extraction. However, resampling also could impact feature extraction as well. Thus, the impacts of voxel size discrepancy and resampling still need to be assessed and compared in the future.

## Methods

### Data collection

In this study, we randomly selected 247 abdominal patients who got abdominal CT examinations at Shanghai Zhongye Hospital. The CT images of the selected patients were exported to an auto-segmentation system named DeepViewer^22^ to segment abdominal ROIs. The segmented ROIs respectively were liver, bowel, left kidney, right kidney, and spinal cord. The abdominal CT images and ROIs of each patient were converted from DICOM to Nifti using dcmstruct2nii^23^ so as to generate the CT and mask data for radiomics feature extraction. The scan voltage was set as 100 kV, and tube current modulation was used.

### Radiomics feature extraction

The Pyradiomics module^24^ was applied to extract radiomics features from each patient’s CT and ROIs. The main procedures included image preprocessing, feature calculation, and feature selection. In the first step, the spatial resolution of the CT and masks for each patient were resampled to (1, 1, 5) to standardize radiomics feature calculation via the Pyradiomics parameter “resamplePixelSpacing”. Data augmentation^25–27^ was performed to enhance the robustness of the SVR model. Data augmentation involved slight shifts and rotations. Zooming was excluded as it could potentially alter the patients’ dosimetric properties. In the second step, 107 radiomics features were extracted for each organ of the patient without using filters.

The radiomics features could be divided into 7 types including Gray Level Co-occurrence Matrix, First Order Statistics, Neighboring Gray Tone Difference Matrix, Gray Level Dependence Matrix, Gray Level Run Length Matrix, Shape-based, and Gray Level Size Zone Matrix. Those features reveal the ROIs’ correlation, homogeneity, contrast, intensity distribution, etc., and were used as input data for training the SVR model. In the third step, the f-regression function the scikit-learn module was used to select the relevant features to avoid overfit^28^ and to enhance the robustness of the SVR model. The main hypotheses of the F-test—including independence, linearity, homoscedasticity, and normality of errors—should ideally be respected for all radiomic features to ensure the validity of the test. A p-value threshold was not explicitly used to select the relevant features; instead, the top 30 features were selected based on their ranking by the F-statistic. The feature extraction and selection were performed with double AMD EPYC 7551 CPUs in the Anaconda 3^29^ environment.

### Reference organ dose calculation

Based on the CT image and masks of each patient, the reference organ doses were calculated by GGEMS, a GPU-based MC particle transport code that can address complex geometries, heterogeneous materials, and multiple radiation sources (photons and electrons)^30^. GGEMS could process MC simulation much faster than CPU-based MC simulation code almost without sacrificing organ dose calculation accuracy. For each patient, the GPU-calculated organ doses were used as the reference organ doses when training the SVR model to predict the organ doses of the liver, stomach, bowel, left kidney, right kidney, and spinal cord. The GPU-based MC simulations were performed with two Nvidia RTX4090 graphics cards to obtain the slice-wise dose distribution of each patient with less than 2% error in each voxel. Auto-tube current was taken into account for MC dose calculation.

### SVR prediction model

This study adopted the trained SVR regression model to reflect the relationship between the input radiomics features and reference organ doses, and predict personalized abdominal organ doses accurately. SVR is a supervised learning algorithm that is used to predict continuous values^31–33^. SVR uses the same principle as support vector machine (SVM), which are classifiers that find the optimal hyperplane that separates two classes of data. Instead of finding a hyperplane that maximizes the margin between the classes, SVR finds a hyperplane that minimizes the error between the predicted and actual values, while allowing some tolerance for deviation.

The performance of the model was assessed by RRMSE, APE, MAPE, and R^2^ on the test set for the ROIs including the liver, stomach, bowel, left kidney, right kidney, and spinal cord. The robustness of the model was verified by randomly assigning patient samples to the train and test sets while keeping the proportion of the two sets as 0.8:0.2 and comparing the regression metrics of different patient sample assigning strategies. The SVR was trained and tested using two Nvidia GeForce RTX4090 GPUs and two Nvidia GeForce RTX3080ti GPUs in the Anaconda environment. Figure 1 shows the General flowchart of training and testing the SVR-based abdominal organ dose prediction model.

**Figure 2:**
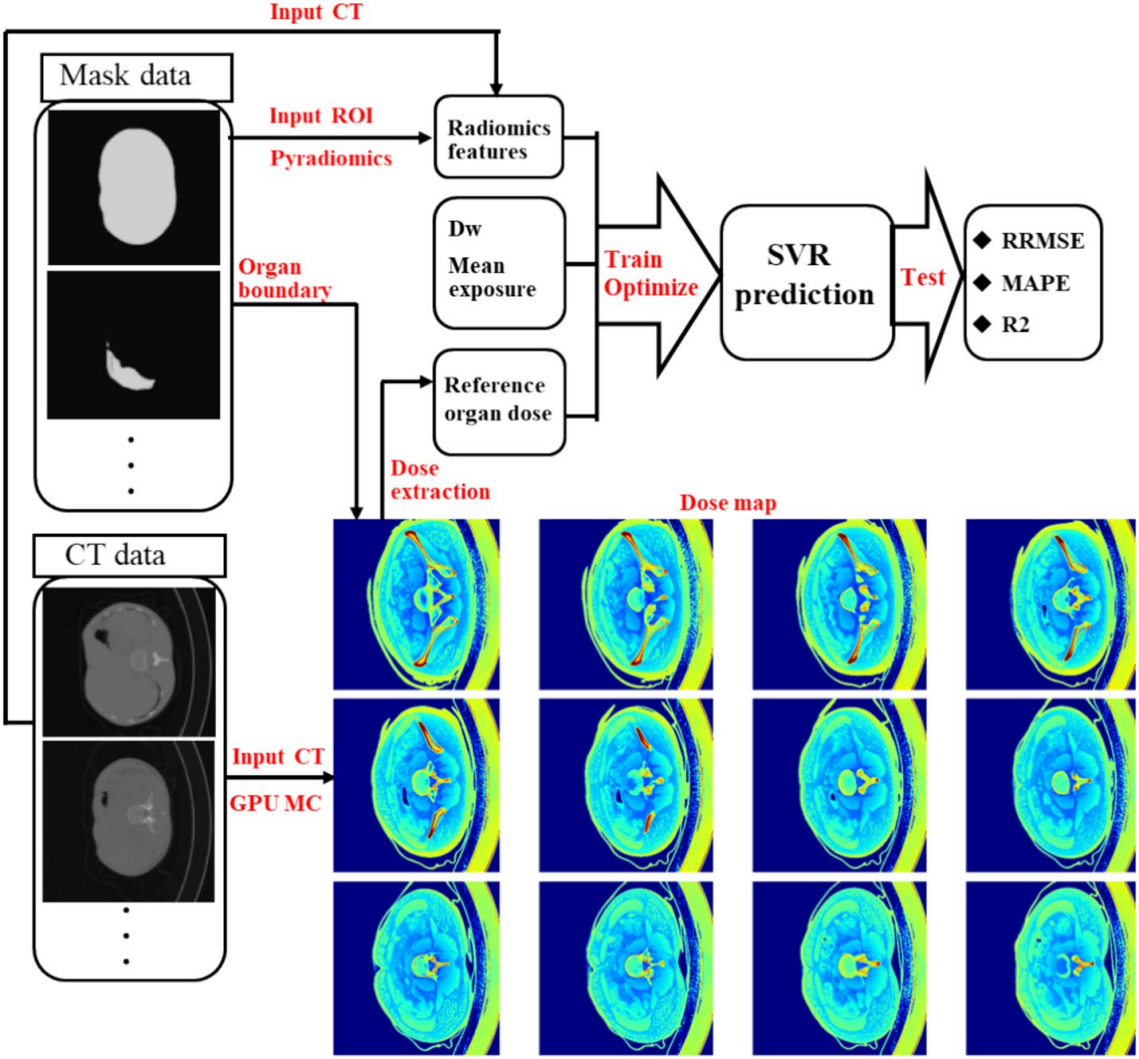
General flowchart of preparing input data plus training and testing the SVR-based abdominal organ dose prediction model.

### Regression metrics for the dose prediction

For assessing the performance of abdominal organ dose prediction, we adopted the regression metrics of RMSE, MAPE, and R^2^. RMSE is the root mean squared error. It measures how large the root mean squared error. A lower RMSE indicates a better fit. RMSE can be calculated as:

1 $$RRMSE=\sqrt{\frac{1}{n}\sum_{i=1}^{n}({y}_{i}-{\widehat{y}}_{i}{)}^{2} }\times 100\text{\%}$$

where n is the number of patients, ${y}_{i}$ is the reference organ dose, and ${\widehat{y}}_{i}$​ is the predicted organ dose. MAPE is the average of the absolute percentage differences between the actual and predicted values. It measures how close the predictions are to the actual values in terms of percentage, without considering the direction of the error. The formula can be expressed as:

2 $$MAPE=\frac{1}{n}\sum_{i=1}^{n}\mid \frac{{y}_{i}-{\widehat{y}}_{i}}{{y}_{i}}\mid \times 100\text{\%}$$

R^2^ is the proportion of the variance in the output variable that is explained by the input variables. It measures how well the regression model fits the data. A higher R^2^ indicates a better fit. The formula is:

3 $$R{2} = 1 - \frac{{\mathop \sum \nolimits_{i = 1}^{n} (y_{i} - \hat{y}_{i} )^{2} }}{{\mathop \sum \nolimits_{i = 1}^{n} (y_{i} - \overline{y})^{2} }}$$

## Data Availability

Data availability Data sets generated during the current study are available from the corresponding author on reasonable request.
